# Large language model-generated versus teacher-written objective structured clinical examination stations for medical students: a blinded comparative pilot study

**DOI:** 10.3352/jeehp.2026.23.9

**Published:** 2026-05-26

**Authors:** Piotr Szychowiak, Jonathan Wong-So, Hélène Messet, Mélanie Faure, Isaure Breteau, Simon Jamard, François Barbier, Maxime Desgrouas

**Affiliations:** 1 Médecine Intensive Réanimation, Centre Hospitalier Universitaire d’Orléans, Orleans, France; 2 Réanimation Chirurgicale, Centre Hospitalier Régional Universitaire de Tours, Orleans, France; 3 Service des Maladies Infectieuses et Tropicales, Centre Hospitalier Régional Universitaire de Tours, Orleans, France; Hallym University, Korea

**Keywords:** Artificial intelligence, Clinical competence, Educational measurement, Medical education, France

## Abstract

Developing objective structured clinical examination (OSCE) stations is time-consuming for medical teachers. We aimed to evaluate the ability of a large language model (LLM) to generate ready-to-use OSCE stations. Five OSCE stations generated by the LLM GPT-4o were evaluated by 7 expert assessors using a 5-point Likert scale and compared with 5 teacher-written stations targeting similar learning objectives. A station was considered to be of good quality if most assessors responded “agree” or “strongly agree” to the statement “The station is good enough to be used by students.” All teacher-written stations were rated as being of good quality, compared with only one GPT-4o-generated station. The LLM produced adequate clinical scenarios when reference knowledge was provided and tasks were clearly ordered, but it failed to generate reliable assessment grids. Careful review by teachers remained essential. GPT-4o failed to consistently produce fully ready-to-use OSCE stations.

## Graphical abstract

[Fig f4-jeehp-23-09]

## Background/rationale

Constructing objective structured clinical examination (OSCE) stations is time-consuming for medical teachers, as each station must be carefully written, reviewed, and tested to ensure its quality. Any means of reducing the time required to produce valid OSCE stations could increase the number of stations available to students, facilitate the creation of station databases, and ultimately enhance training. Large language models (LLMs) are increasingly used in medical education and have demonstrated the ability to perform OSCEs, simulate standardized participants, and assess conversational OSCEs [1-3]. In contrast, their application to the generation of highly complex educational content, such as OSCE stations, remains largely unexplored [4].

## Objectives

We hypothesized that the GPT-4o LLM could support medical teachers in producing ready-to-use OSCE stations. The primary objective of this study was to assess the quality of GPT-4o-generated OSCE stations compared with similar stations written by trained medical teachers.

## Ethics statement

No institutional review board approval was required.

## Study design and setting

The study used the METRICS checklist (Supplement 1, Table S1) to guide the design and reporting of the use of generative artificial intelligence in medical education [5]. Five GPT-4o-generated OSCE stations were blindly compared with 5 “mirror” stations written by a medical teacher and targeting the same learning objectives. The study followed 4 sequential steps: prompt engineering, station writing by the medical teacher, station generation by GPT-4o (up to 3 attempts), and blinded assessment of the stations. Writing and generation times were recorded. The study involved 1 assistant professor responsible for prompt engineering and for writing and generating the OSCE stations, as well as 7 academic physicians (1 full professor and 6 assistant professors). To ensure consistency in evaluation, each teacher was selected based on training and experience in OSCE development and evaluation; all were certified after completing the French national OSCE evaluator training course (https://formation.uness.fr/formation/course/view.php?id=20081). The paid version of GPT-4o was selected because of its availability and because it outperformed other LLMs in prior evaluations [6].

## Data sources/measurement

All data were collected prospectively. Compliance with quality standards for OSCE stations was evaluated using 5-point Likert scales (strongly disagree, disagree, neither agree nor disagree, agree, strongly agree) (Supplement 1, Table S2) based on the French guidelines for medical OSCE assessors (OSCE vademecum: https://formation.uness.fr/formation/pluginfile.php/332080/mod_resource/content/5/Vademecum%20ECOS.pdf) and a published assessment grid [7]. Each teacher independently evaluated each station using the Likert-scale–based assessment grid. Individual item ratings were collected at the teacher level and analyzed across teachers for each item, with results presented as the distribution of Likert-scale responses by station type (GPT-generated vs. teacher-written). The primary endpoint was the ready-to-use quality of the OSCE stations, which was defined at the station level based on a majority rule: a station was considered ready-to-use if at least 4 of the 7 teachers responded “agree” or “strongly agree” to the statement “The station is good enough to be used by students.” This threshold was defined pragmatically to reflect consensus among expert assessors, consistent with common practices in educational evaluation based on aggregated expert judgment. Assessment of OSCE station quality was blinded to the origin of the stations (GPT-4o-generated vs. teacher-written).

## Statistical methods

Categorical variables are presented as frequencies and percentages, and continuous variables are presented as medians with interquartile ranges (IQRs). Fisher exact tests and paired Wilcoxon signed-rank tests were used as appropriate. Inter-rater agreement was assessed using the intraclass correlation coefficient (ICC). Analyses were performed using R software ver. 4.4.1 (The R Foundation for Statistical Computing).

## Main results

### Prompt engineering

The large language model used was GPT-4o (OpenAI), accessed in August 2024 via the ChatGPT application. Default model parameters were applied. Prompt engineering was performed by a single medical teacher (M.D.) using a progressive increase in prompt complexity, ranging from a simple instruction (“write an OSCE station about septic shock for medical students”) to a 3,000-word prompt. The final prompt was structured into 4 components: instructions regarding tags, context and general objectives, review of the reference course, and step-by-step generation of each section (general information, clinical situation, instructions for the standardized participant, iconography, and assessment grid). It assigned GPT-4o the role of a medical teacher and included reference teaching material on sepsis and septic shock, ordered tasks with chained instructions, examples, and structured tags (“context,” “task,” “instruction,” and “refine”) designed to optimize the LLM’s output. A translation of the prompt is available in Supplement 1. The final version of the prompt consistently generated OSCE stations on septic shock that included all expected sections (clinical situation, learning objectives, standardized participant script, laboratory results, and evaluation grid), regardless of content relevance. This prompt was validated on other medical topics, which were not included in the final evaluation, to ensure reliability. Prompt calibration required a total of 24 hours of work.

### Generation of the stations

**Fig. 1.**
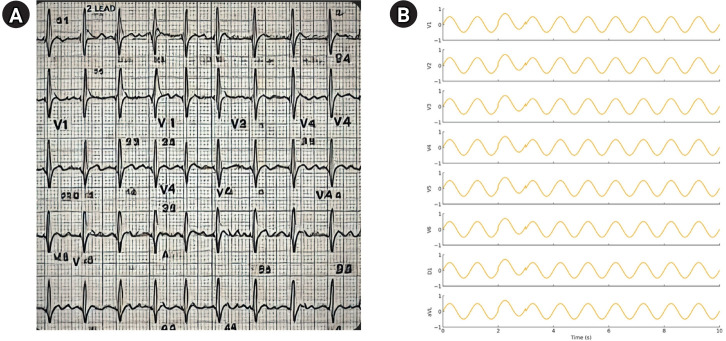
(A, B) Electrocardiogram tracings generated by GPT-4o for objective structured clinical examination station number 1. (A) Generated using the prompt: “generate a typical electrocardiogram tracing of an anterior myocardial infarction.” (B) Generated using the prompt: “generate an electrocardiogram tracing with ST-segment elevation in leads V1 to V6, I, and aVL.” Both tracings failed to produce a clinically correct electrocardiogram.

**Fig. 2.**
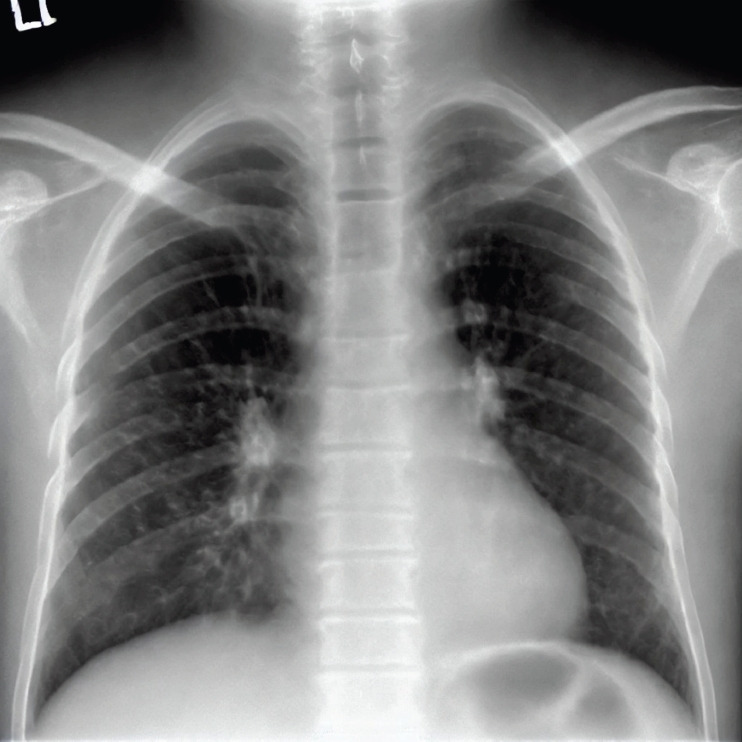
Chest X-ray generated by GPT-4o for objective structured clinical examination station number 2. Generated using the prompt: “generate a chest X-ray showing a right lower lobe pneumonia.” GPT-4o produced an image resembling a chest X-ray but failed to depict alveolar consolidation in the right lower lobe. Notably, several anatomical abnormalities can be observed, including an additional right rib, vertebral compression, and apparent absence of spinous processes.

The prespecified characteristics of the 5 OSCE stations were based on the national second-cycle knowledge item list and are presented in Table 1. Writing and generation times are also reported in Table 1. The total time required to generate an OSCE station using GPT-4o comprised model output time, formatting according to the OSCE template (GPT-4o was unable to generate content directly in .docx format during prompt engineering, despite being provided with a corresponding .docx template), and proofreading without major content modification. For paired OSCE stations, the median time required for manual writing was 54 minutes (IQR, 53–55 minutes), compared with 24 minutes (IQR, 22–31 minutes) using GPT-4o, yielding a median paired time saving of 29 minutes (IQR, 20–33 minutes) (P=0.06). GPT-4o successfully generated relevant laboratory results, including antibiotic susceptibility testing and arterial blood gas analyses, but failed to produce appropriate electrocardiogram tracings or chest X-ray images (Figs. 1, 2). Electrocardiogram tracings were replaced with real tracings for the evaluation, whereas the chest X-ray was retained. In addition, GPT-4o failed to generate an OSCE station involving a standardized patient for OSCE station 3 and consistently produced a station involving a standardized healthcare professional instead across all 3 attempts.

## Key results

**Fig. 3.**
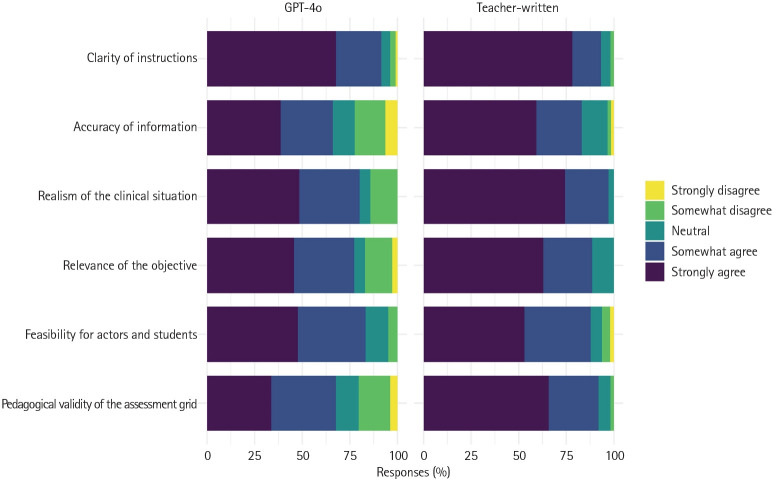
Compliance of objective structured clinical examination (OSCE) stations with OSCE vademecum. Distribution of Likert-scale responses of teachers by station type (GPT-4o-generated vs. teacher-written). “Clarity of instructions” corresponded to questions 1 to 3 of the station assessment grid. “Accuracy of information” included questions 6 and 8. “Realism of the clinical situation” was assessed by question 4, while “Relevance of the objective” was assessed by question 5. “Feasibility for actors and students” encompassed questions 7 and 15. Finally, “Pedagogical validity of the assessment grid” included questions 9 to 14. Details of the assessment grid are provided in Supplement 1.

One of the 5 GPT-4o-generated OSCE stations was considered ready-to-use, compared with all 5 teacher-written stations (Table 2). Discrepancies between learning objectives and evaluation grids and lack of accuracy were the main issues identified by teachers in the GPT-4o-generated OSCE stations. GPT-4o occasionally failed to appropriately align the assessment grid with the clinical data or the instructions provided to standardized patients. No concerns were raised regarding the generated laboratory results. All assessors noted the inappropriateness of the chest X-ray. Hallucinations generated by GPT-4o may have occurred, as some assessment grid items or learning objectives were not aligned with the OSCE vademecum. Details of compliance with the OSCE vademecum for each station are presented in Fig. 3. Inter-rater agreement was moderate, with an ICC of 0.65 (95% confidence interval, 0.55–0.73).

## Interpretation

Our study compared 5 GPT-4o-generated OSCE stations with 5 teacher-written OSCE stations targeting identical learning objectives. Only one GPT-4o-generated OSCE station was deemed ready-to-use for student training, compared with all 5 teacher-written stations. Although most clinical situations were considered realistic and the instructions provided to students and standardized participants were considered clear, lack of realism in some stations, the presence of hallucinations, and low-quality assessment grids prevented most GPT-4o-generated stations from being considered ready-to-use. Interestingly, station 3, for which GPT-4o failed to generate a scenario involving a standardized patient, was paradoxically rated higher than both the corresponding teacher-written station and the other GPT-4o-generated stations. This finding may be explained by the fact that the teacher-written station was considered too difficult for students, particularly with regard to interpretation of the antibiogram, and lacked realism because it required explanation of an antibiogram to a standardized patient. Furthermore, because this station was the most difficult to generate due to repeated non-compliance with the instructions, 3 attempts were required, which may partly explain why it performed slightly better than the other 4 GPT-4o-generated cases. The inability to generate accurate medical imaging limited the range of clinical situations that could be realistically produced. The time saved during station generation was not significantly different from that required for manual OSCE writing and may be offset by the extensive revision needed for GPT-4o-generated stations.

## Comparison with previous study

Another study compared 3 prompting strategies for generating OSCE stations in the field of digital health education [4]. Their simulated-agent GPT strategy—similar to our prompting approach but relying on sequential generation with provision of intermediate outputs before each subsequent step—achieved high compliance with the prompt, except for the assessment grid. Consistent with our findings, they reported difficulties for GPT-4o in consistently producing observable, distinct, and sufficiently detailed assessment items that would allow reproducible grid completion. Although they did not report hallucinations, they identified inconsistencies such as inaccurate or missing information and implicit answers. Their decision to split tasks between GPT agents resembles our strategy of adding tags to different tasks to distinguish contextual explanations, task structure, specific outputs, and refinement of previous generations [8]. Another study focusing on artificial intelligence-generated medical cases—which are comparable to OSCE stations given their complexity—also highlighted the strong potential of such tools in medical education while emphasizing the need to improve case realism, consistency, and relevance [9]. Neither of these studies estimated time savings or compared the quality of generated cases with standard teacher-written cases.

## Limitations/generalizability

Our study has several limitations. The success rate for generating ready-to-use OSCE stations was lower in our study (1 out of 5) than in previous studies [4,9]. This difference may be explained by our prompting strategy, which aimed to generate a complete OSCE station in a single output, whereas previous studies relied on sequential, step-by-step case generation. As inputs become longer, LLMs tend to use the available information less consistently [10]. Furthermore, prompt engineering, station generation, and station writing were all performed by the same teacher, which may have introduced bias. The performance of LLMs is rapidly evolving, and future improvements may affect the reproducibility and comparability of results obtained using identical prompts. Our a priori objective was not to modify the generated content and to obtain a fully “ready-to-use” OSCE station in each case, which may have been overly ambitious. Rather than acting as a complete substitute for OSCE writing, LLMs may be better suited as tools for saving time on specific components of OSCE construction, such as drafting clinical scenarios or standardized patient scripts. However, GPT-4o outputs are not reproducible, even when the same prompt is used repeatedly.

## Suggestions

GPT-4o and future versions may warrant further exploration as supportive tools, for example by generating clinical scenarios aligned with predefined learning objectives while using assessment grids established beforehand by expert medical teachers. Such an approach may reduce the risk of inconsistency between the clinical case and the assessment grid while also saving time.

## Conclusion

GPT-4o failed to consistently produce fully ready-to-use OSCE stations. However, it may warrant further evaluation as a supportive and time-saving tool for generating specific components of OSCE stations under the supervision of medical teachers.

## Figures and Tables

**Figure f4-jeehp-23-09:**
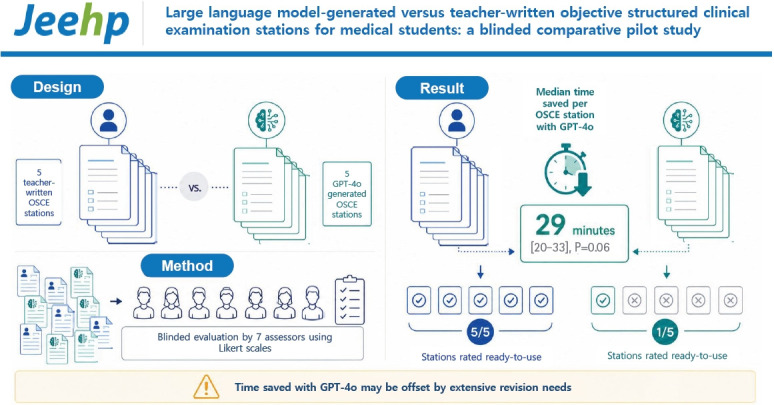


**Table 1.** Description of OSCE stations and their production time by teachers or GPT-4o

OSCE no.	Item	Initial clinical situation	Learning domain	Standardized participant	Production time (min)	Time saving (min)^a)^
1	Acute coronary syndrome	185^b)^: Performance and interpretation of an electrocardiogram	Appropriate management strategy	None	Teacher: 38 GPT-4o: 18^c)^	20
2	Severe community-acquired pneumonia	160^b)^: Acute respiratory distress	Vital emergency	Standardized healthcare worker	Teacher: 55 GPT-4o: 22^c)^	33
3	Emergency antibiotic therapy	187^b)^: Multidrug-resistant bacteria on antibiogram	Synthesis of diagnostic test results	Standardized patient	Teacher: 54 GPT-4o: 48^c)^	6
4	Management of errors and complaints, therapeutic adverse events	331^b)^: Identification of a therapeutic adverse event or medical error	Interprofessional communication	Standardized healthcare worker	Teacher: 53 GPT-4o: 24^c)^	29
5	Acid–base balance disorders and hydroelectrolytic disturbances	13^b)^: Nausea/vomiting	Diagnostic strategy	Standardized patient	Teacher: 73 GPT-4o: 31^c)^	42

OSCE, objective structured clinical examination.

^a)^Median paired time saving of 29 minutes (interquartile range, 20–33 minutes), P=0.06, using the paired Wilcoxon signed-rank test. ^b)^National learning objective codes. ^c)^The total time required to generate an OSCE station using GPT-4o comprised model output time, formatting according to the OSCE template, and proofreading.

**Table 2.** Quality evaluation of teacher-written and GPT-4o-generated OSCE stations rated by 7 assessors

OSCE no./OSCE design	“Ready-to-use” OSCE evaluation (n=7)	P-value
1		0.1
Teacher	6/7	
GPT-4o	2/7	
2		0.07
Teacher	7/7	
GPT-4o	3/7	
3		1
Teacher	4/7	
GPT-4o	5/7	
4		0.02
Teacher	7/7	
GPT-4o	2/7	
5		0.02
Teacher	7/7	
GPT-4o	2/7	

A station was considered to be of good quality if at least 4 of the 7 assessors responded “agree” or “strongly agree” to the statement “The station is good enough to be used by students.” OSCE, objective structured clinical examination.

## Supplementary materials

Supplement 1. METRICS checklist, Likert scale and prompt.

Supplement 2. Audio recording of the abstract.
