# Supplementary material for: Large language model-generated versus teacher-written objective structured clinical examination stations for medical students: a blinded comparative pilot study
Source: J Educ Eval Health Prof. 2026 May 26;23:9. doi: 10.3352/jeehp.2026.23.9 (PMC13287236; doi:10.3352/jeehp.2026.23.9)
Supplement: Supplementary file 2 — Supplement 1. METRICS checklist, Likert scale and prompt. [file jeehp-23-09-suppl1.docx]

**Supplement 1.** Supplementary material

**Table S1.** METRICS checklist

| METRICS item | Issue considered in each item | Response |
| --- | --- | --- |
| Model | What is the model of the generative AI tool used for generating content, and what are the exact settings for each tool? | GPT-4o (OpenAI) through Application Programming Interface (API). Prompt is provided below. |
| Timing | When is the generative AI model tested exactly and what are the duration and timing of testing? | OSCE stations were generated in August 2024. Prompt calibration required a cumulative total of 24 hours of work. The time required to generate OSCE stations is reported in Table 1. |
| Count | What is the count of queries executed (sample size)? | Five OSCE stations were generated using a single prompt. For each OSCE station, up to 3 generation attempts were allowed. |
| Specificity of the prompt or language | How specific are the exact prompts used? Were those exact prompts provided fully? Did the authors consider the feedback and learning loops? How specific are the language and cultural issues considered in the generative AI model? | The prompt is provided below. It was written in French, using appropriate medical terminology, by a single medical teacher during the calibration step described in the Methods. No feedback or iterative learning loop was used. |
| Evaluation | What is the exact approach used to evaluate the content generated by the generative AI-based model and is it an objective or subjective evaluation? | Evaluation was performed by 7 medical teachers (academic physicians, 1 full professor, 6 assistant professors) at the station-level using a 5-point Likert scale comprising 16 items assessing compliance with OSCE vademecum guidelines. |
| Individual factors | Is there any individual subjective involvement in generative AI content evaluation? If so, did the authors describe the details in full? | Assessors were blinded to the AI-generated origin of the OSCE stations and were informed that they were evaluating OSCE stations written by students for a training database. |
| Transparency | How transparent are the data sources used to generate queries for the generative AI-based model? | Prompts were combined with the institutional OSCE template, national OSCE vademecum guidelines, and reference learning objectives from the Collège des Enseignants de Médecine Intensive Réanimation. |
| Range | What is the range of topics tested and are they intersubject or intrasubject with variability in different subjects? | The 5 OSCE stations addressed 5 distinct medical topics across 5 specialties, with different initial clinical situations and learning domains. Each station included either 1 standardized patient or none. |
| Randomization | Was the process of selecting the topics to be tested on the generative AI-based model randomized? | Topics of the generated OSCE were arbitrarily selected a priori by a medical teacher but were not used during the calibration step. |

AI, artificial intelligence; OSCE, objective structured clinical examination.

**Table S2.** Likert scale used for OSCE station evaluation (based on OSCE vademecum guidelines)

| Item | Strongly disagree | Disagree | Neither agree nor disagree | Agree | Strongly agree |
| --- | --- | --- | --- | --- | --- |
| Q1. The general information of the station is complete | Strongly disagree | Disagree | Neither agree nor disagree | Agree | Strongly agree |
| Q2. The material required for the station is adequately detailed | Strongly disagree | Disagree | Neither agree nor disagree | Agree | Strongly agree |
| Q3. The clinical scenario is clear and concise | Strongly disagree | Disagree | Neither agree nor disagree | Agree | Strongly agree |
| Q4. The clinical scenario is appropriate | Strongly disagree | Disagree | Neither agree nor disagree | Agree | Strongly agree |
| Q5. The instructions to the student (what you must/must not do) are appropriate | Strongly disagree | Disagree | Neither agree nor disagree | Agree | Strongly agree |
| Q6. The instructions to the standardized patient or standardized healthcare staff are sufficient | Strongly disagree | Disagree | Neither agree nor disagree | Agree | Strongly agree |
| Q7. The instructions to the standardized patient are suitable for a non-medical actor | Strongly disagree | Disagree | Neither agree nor disagree | Agree | Strongly agree |
| Q8. The iconography/additional examinations are appropriate for the station | Strongly disagree | Disagree | Neither agree nor disagree | Agree | Strongly agree |
| Q9. The clinical skills section of the assessment grid consists of items observable by the examiner | Strongly disagree | Disagree | Neither agree nor disagree | Agree | Strongly agree |
| Q10. The clinical skills items in the assessment grid consist of distinct elements or are precisely grouped (A and B, A or B, completed if >2 items cited, etc.) | Strongly disagree | Disagree | Neither agree nor disagree | Agree | Strongly agree |
| Q11. The clinical skills items in the assessment grid are dichotomous (performed/not performed) | Strongly disagree | Disagree | Neither agree nor disagree | Agree | Strongly agree |
| Q12. The clinical skills section of the assessment grid is adequately detailed for the examiner (neither too much nor too little) | Strongly disagree | Disagree | Neither agree nor disagree | Agree | Strongly agree |
| Q13. The clinical skills section of the assessment grid is discriminative in distinguishing higher- and lower-performing students | Strongly disagree | Disagree | Neither agree nor disagree | Agree | Strongly agree |
| Q14. The communication and attitudes section of the assessment grid is appropriate | Strongly disagree | Disagree | Neither agree nor disagree | Agree | Strongly agree |
| Q15. The OSCE station is feasible within the allocated time (8 minutes) | Strongly disagree | Disagree | Neither agree nor disagree | Agree | Strongly agree |
| Q16. The station is good enough to be used by students | Strongly disagree | Disagree | Neither agree nor disagree | Agree | Strongly agree |

Initial Likert scale was in French.

OSCE, objective structured clinical examination.

**Translation of the prompt used to generate OSCE**

I will give you instructions using labels in parentheses to specify what is expected.

(context) indicates the context in which you should position yourself.

(task) indicates what you must do, and (instruction) how you must do it.

Finally, (refine) asks you to check and correct your errors.

(ss) indicates a new sub-section within a section.

⸻

(context) Act as an academic physician at a medical school teaching second-cycle medical students.

(instruction) I want you to write an Objective Structured Clinical Examination (OSCE) station vignette **with/without a standardized patient/standardized healthcare staff,** based on the initial situation **“initial situation”,** with the learning domain **“learning domain”,** and with the main objective of the station being **“main objective of the station”.**

(context) The OSCE is a tool used to assess students’ clinical performance in a simulated environment. It is a combined assessment of knowledge, skills, and professional attitudes. Its aim is to approximate student competence by judging performance in a planned and structured way through a clinical scenario lasting 8 minutes.

(context) Depending on the OSCE scenario, the student may be alone (OSCE without actor), facing a simulated patient (OSCE with standardized patient), or facing a healthcare professional such as a physician, nurse, or nursing assistant (standardized healthcare professional), but never all 3 situations at the same time.

(instruction) Carefully take this information into account for the rest of the task.

⸻

Steps:

1- (task) Review the content of the reference course **“reference course”**, which includes all the knowledge that students are expected to have.

a. (instruction) All information you write for the vignette must come exclusively from this document.

b. (instruction) Knowledge is classified at the beginning of the document by rank. You may only use Rank A and Rank B knowledge; no Rank C knowledge is allowed.

c. (instruction) The document contains several vignettes and clinical situations that you may draw inspiration from, but you must not copy them verbatim into the OSCE vignette.

2- (task) Write the “General Information” section of the OSCE vignette in table format.

(instruction) The expected sub-sections are listed below with details and examples:

a. (ss) Station name:

(instruction) Title/name of the station, which must be generic and should not reveal too much information about the content or the questions that will be asked of students.

b. (ss) Level:

(instruction) Choose among: MM1, MM2, MM3 (from the easiest to the most difficult level, depending on the content you produce).

c. (ss) Knowledge:

(instruction) Choose among: Rank A, Rank B, or Ranks A and B.

d. (ss) Learning domain(s):

(instruction) Each station must assess 1 main learning domain, with an optional secondary domain if needed, which must remain minor.

(instruction) Choose 1 domain from the following list:

i. History taking

ii. Synthesis of paraclinical examination results

iii. Diagnostic strategy

iv. Education/Prevention

v. Appropriate management strategy

vi. Life-threatening emergency

vii. Patient disclosure/Information

viii. Interprofessional communication

ix. Physical examination

x. Iconography

xi. Procedure

e. (ss) Initial situation:

(instruction) The one provided at the beginning of the instructions.

f. (ss) Discipline:

(instruction) Choose among medical and surgical specialties: Cardiology, biology, intensive care, public health, etc.

g. (ss) Item:

(instruction) The item(s) defined at the beginning of the provided reference course.

Example: item 332 – Shock.

h. (ss) Station objectives:

i. (instruction) Define the expected learning objectives of the station. These are instructions for the examiner to facilitate evaluation of the student.

ii. (instruction) You may select up to 3 objectives from Rank A and Rank B items provided at the beginning of the reference course.

Example: the student must be able to diagnose a state of shock; the student must be able to explain the initial management to the nurse so that the examiner can assess their ability to manage an emergency; etc.

iii. (instruction) Do not include objectives related to history taking or explaining information to the patient if there is no standardized patient (SP) in the vignette.

iv. (instruction) Do not include objectives related to explanation or communication with a nurse or physician if there is no standardized healthcare professional (SHP) in the vignette.

i. (ss) Actor:

(instruction) Specify: None; Standardized Patient (SP); Standardized Healthcare Professional (SHP), according to the instructions given above.

(instruction) Warning: only one actor is allowed per station; there must never be both an SP and an SHP at the same time.

j. (ss) Required material:

(instruction) Specify any material required depending on the station objectives.

Examples: None; ECG ruler; blank prescriptions; sick leave form; death certificate; chest X-ray showing a middle lobe pneumonia; procedural mannequin for digital rectal examination; blood pressure cuff; etc.

i. (instruction) Material always required: scrap paper, pen. Clinical vignette available in the room. Stopwatch/clock.

ii. (instruction) Only include material that is relevant and useful for the objectives listed in the “Station objectives” sub-section.

iii. (instruction) Warning: OSCEs are not simulation sessions; there will never be simulated acts except in procedural stations using mannequins. In most cases, it is unnecessary to include infusion equipment, medications, or invasive procedure equipment.

3- (refine) Ensure that each sub-section of the “General Information” section is completed and coherent.

4- (task) Write the “Vignette and instructions” section in table format.

(instruction) The expected sub-sections are listed below with details and examples:

a. (ss) Situation:

(instruction) Describe the clinical situation.

i. (instruction) By default, the student is always an intern.

ii. (instruction) Describe the initial situation clearly and concisely, providing useful and necessary information to achieve the objectives of the vignette. Some information may not be provided initially and may be given by the standardized patient depending on the student’s questions.

b. (ss) You must:

(instruction) List the station objectives to be achieved within the allotted time.

Examples: you must take a history; you must interpret the chest X-ray; you must verbally present your main diagnostic hypothesis to the examiner; etc.

i. (instruction) Maximum of 3 objectives.

ii. (instruction) These objectives must be consistent with those in the “General Information” section.

iii. (instruction) You may select up to 3 objectives from Rank A and Rank B items provided at the beginning of the reference course.

iv. (instruction) Below are the main objectives that may be requested of a student and that you may adapt based on Rank A and B items:

• take a patient history

• perform a physical examination on a standardized patient or mannequin

• perform an oral or written handover

• perform a technical procedure

• interpret clinical photographs, laboratory results, imaging, etc.

• deliver bad news or explain indications, risks, benefits, alternatives, and potential complications of a therapeutic plan, procedure, or invasive act

• propose a diagnostic and/or therapeutic strategy

• conduct an educational/preventive intervention

• interact with a standardized healthcare professional

c. (ss) You must not:

(instruction) List potential objectives that could be coherent but are not expected in this vignette and that the student might attempt to perform.

Examples: you must not examine the patient; you must not write a prescription; etc.

5- (refine) Ensure that each sub-section of the “Vignette and instructions” section is completed and coherent with the other sections and sub-sections.

6- (task) Write the “Instructions to the actor” section.

(instruction) Describe the situation as precisely as possible for the standardized patient/standardized healthcare professional.

(instruction) This section must only be present if the OSCE vignette includes an actor.

(instruction) The expected sub-sections are listed below with details and examples:

a. (ss) Scenario reminder:

i. (instruction) Technical/clinical objectives: brief reminder of the scenario’s objective for the learner

ii. (instruction) Care setting: example: emergency department

iii. (instruction) Position of the SP and candidate: example: the SP is seated facing the student

b. (ss) Mindset and behavior:

i. (instruction) Mindset: example: you are anxious; you are angry; etc.

ii. (instruction) Attitude during the interview: example: you speak little; you hold your abdomen because you are in pain; your hands are shaking; etc.

iii. (instruction) Signs to simulate: example: painful facial expression; word-finding difficulty; double vision; etc.

c. (ss) Additional data:

(instruction) Indications/instructions: specify whether documents must be systematically handed to the candidate and in what order. It is recommended not to include dates or identities on documents, but rather “the day before,” “1 month ago,” “Dr Name in City.”

d. (ss) Opening sentence:

(instruction) Opening phrase to specify if needed and if the actor plays an SP or SHP able to communicate.

Example: “Hello Doctor, my general practitioner asked me to come and see you because I have lost 5 kg over the past 3 months.”

e. (ss) Identity:

(instruction) Specify the main scripted identity elements of the SP: last name, first name, height, weight.

Example: Mr. C, 65 years old, 1.65 m tall, weighing 54 kg.

f. (ss) Socio-professional context and leisure activities:

i. (instruction) Personal situation: example: married, 3 children.

ii. (instruction) Living conditions: example: lives in an apartment with an elevator; limited autonomy with assistance for bathing; etc.

iii. (instruction) Occupation: specify whether the patient is employed and their profession if relevant to the scenario.

iv. (instruction) Tobacco/Alcohol: specify alcohol and tobacco use and quantify if relevant.

Example: active smoker, 1 pack per day for 30 years; 3 glasses of wine with each meal; etc.

g. (ss) Medical history and treatments:

i. (instruction) Personal medical history: specify medical and surgical history; specify allergies. Do not give dates but time intervals.

Example: appendectomy at age 8; nephrectomy 2 years ago; etc.

ii. (instruction) Family history: specify if relevant to the scenario.

Example: type 1 diabetes in brother and sister.

iii. (instruction) Current medications: list main treatments using INN names. Dosage should only be specified if relevant to the scenario.

Example: paracetamol for headache versus paracetamol 12 grams per day in a drug-induced hepatitis scenario.

h. (ss) Symptoms:

i. (instruction) Symptom description and intensity: specify type, location, intensity, course, aggravating factors, relieving factors, duration, etc. The scenario must be usable at any time, so indicate time intervals rather than dates, unless relevant (example: winter for carbon monoxide poisoning).

Examples: severe transfixing epigastric pain worsened by food intake with a pain score of 8/10; non-bloody watery diarrhea with 6 stools per day for 1 week.

ii. (instruction) Clinical examination/history data: specify associated findings related to the main symptom that the student should seek.

Example: skin rash last month; knee joint swelling 1 year ago; dysuria for 1 week; etc.

iii. (instruction) Conditions for disclosure of additional information: specify whether certain data (information or documents) should only be disclosed by the SP under certain conditions.

Examples: only provide outpatient laboratory results if the student asks whether you have a baseline creatinine; only provide your child’s health record if the student asks whether vaccinations are up to date; only inform the student about a history of dyslipidemia if explicitly asked; etc.

7- (refine) Ensure that each sub-section of the “Instructions to the standardized patient” section is completed and coherent with the “General Information” and “Vignette and instructions” sections and their sub-sections.

8- (task) Create an “Iconography” section containing documents and examination results useful for the vignette, considering what you proposed in the “Vignette and instructions” and “Instructions to the standardized patient” sections.

a. (instruction) Generate laboratory results that are appropriate and coherent with previous sections only if necessary for the vignette.

b. (instruction) Generate an electrocardiogram that is appropriate and coherent with previous sections only if necessary for the vignette.

c. (instruction) Generate imaging that is appropriate and coherent with previous sections only if necessary for the vignette.

d. (instruction) Generate a physician’s letter or the SP’s prescription that is appropriate and coherent with previous sections only if necessary for the vignette.

9- (refine) Ensure that you have produced complete documents and examination results in the “Iconography” section that are coherent with the “Vignette and instructions” and “Instructions to the standardized patient” sections and their sub-sections.

10- (task) Write an “Assessment grid” section containing the assessment grid in table format.

a. (ss) Clinical skills:

i. (instruction) 10 to 15 items of medical expertise (column 1)

ii. (instruction) Dichotomous scale: 1 point if item performed, 0 otherwise (column 2)

iii. (context) The grid is not intended to be exhaustive; it should include only elements relevant for distinguishing higher- and lower-performing students.

(instruction) Items must be dichotomous (performed/not performed), observable, and distinct (1 concept per item).

iv. (instruction) Items in this section should be phrased as: “asks about,” “inquires whether,” “looks for,” “explains to the patient that.”

(instruction) Use short terms understandable by an examiner not specialized in the field.

(instruction) Specify whether elements must all be cited or partially cited to obtain the point using AND/OR conjunctions.

Examples: “looks for the presence of arterial hypertension AND dyslipidemia” relevant for an item “looks for cardiovascular risk factors,” to be grouped in some situations but separated into 2 items in others; “asks about pruritus OR looks for scratch lesions”; “informs the patient of the cancer diagnosis AND specifies localized disease OR absence of metastases”; “inquires about vital signs (credited only if all of the following are requested: Glasgow Coma Scale, blood pressure, heart rate, respiratory rate, SpO₂, temperature).”

v. (instruction) Relevance may be assessed through logical sequencing of tasks.

Example: “proposes initiation of curative anticoagulation WITHOUT WAITING for imaging results.”

vi. (refine) Ensure that the “Clinical skills” sub-section includes only Rank A or B knowledge from the reference course.

vii. (refine) Ensure that the “Clinical skills” sub-section includes 10 to 15 medical expertise items.

viii. (refine) Ensure that no history-taking items are included in the assessment grid if the vignette does not include an SP or SHP.

b. (ss) Communication and attitude:

i. (instruction) 2 to 5 behavioral assessment items, scored from 0 to 1 point per item, in 0.25 increments.

ii. (instruction) Choose from the 13 validated items:

• Ability to listen to the patient/peer (only if vignette includes SP or SHP)

• Ability to ask questions (only if vignette includes SP or SHP)

• Ability to provide information to the patient/caregivers (only if vignette includes SP or SHP)

• Ability to structure/conduct the interview (only if vignette includes SP)

• Non-verbal communication (only if vignette includes SP or SHP)

• Communication with peers (only if vignette includes SHP)

• Ability to cooperate with peers (only if vignette includes SHP)

• Ability to synthesize data

• Ability to structure the physical examination

• Ability to conduct the physical examination

• Ability to plan care

• Ability to propose management

• Ability to perform the technical procedure (only if procedural vignette)

iii. (refine) Ensure that no history-taking items are included in the assessment grid if the vignette does not include an SP or SHP.

iv. (refine) Ensure that the “Communication and attitude” sub-section includes 2 to 5 behavioral assessment items.

11- (refine) Ensure that assessment grid items are coherent with the “Vignette and instructions” and “Instructions to the standardized patient” sections and their sub-sections.

12- (refine) Verify that what you produce is strictly based on the provided reference course.

13- (refine) Verify that you have written all sections indicated by the (task) label and that you have followed all instructions indicated by the (instruction) label.
